# Characterizing Cardiac Function in ICU Survivors of Sepsis: A Pilot Study Protocol

**DOI:** 10.1016/j.chstcc.2024.100050

**Published:** 2024-03

**Authors:** Kevin Garrity, Christie Docherty, Kenneth Mangion, Rosie Woodward, Martin Shaw, Giles Roditi, Benjamin Shelley, Tara Quasim, Philip McCall, Joanne McPeake

**Affiliations:** a Academic Unit of Anaesthesia, Critical Care and Peri-Operative Medicine, University of Glasgow, Glasgow; b School of Cardiovascular and Metabolic Health, University of Glasgow, Glasgow; c Glasgow Royal Infirmary, NHS Greater Glasgow & Clyde, Glasgow; d Imaging Centre of Excellence, Queen Elizabeth University Hospital; NHS Greater Glasgow & Clyde, Glasgow; e University Hospital Crosshouse; NHS Ayrshire and Arran, Crosshouse; f Golden Jubilee National Hospital, NHS Scotland, Clydebank, Scotland; g THIS Institute, University of Cambridge, Cambridge, England

**Keywords:** biomarkers, cardiovascular dysfunction, CMR imaging, critical illness, functional outcomes, heart failure, post-intensive care syndrome, sepsis

## Abstract

**Background:**

Sepsis is one of the most common reasons for ICU admission and a leading cause of mortality worldwide. More than one-half of survivors experience significant physical, psychological, or cognitive impairments, often termed post-intensive care syndrome (PICS). Sepsis is recognized increasingly as being associated with a risk of adverse cardiovascular events that is comparable with other major cardiovascular risk factors. It is plausible that sepsis survivors may be at risk of unidentified cardiovascular disease, and this may play a role in functional impairments seen after ICU discharge.

**Research Question:**

What is the prevalence of myocardial dysfunction after an ICU admission with sepsis and to what extent might it be associated with physical impairments in PICS?

**Study Design and Methods:**

Characterisation of Cardiovascular Function in ICU Survivors of Sepsis (CONDUCT-ICU) is a prospective, multicenter, pilot study characterizing cardiovascular function and functional impairments in survivors of sepsis taking place in the west of Scotland. Survivors of sepsis will be recruited at ICU discharge and followed up 6 to 10 weeks after hospital discharge. Biomarkers of myocardial injury or dysfunction (high sensitivity troponin and N-terminal pro B-type natriuretic peptide) and systemic inflammation (C-reactive protein, IL-1β, IL-6, IL-10, and tumor necrosis factor alpha) will be measured in 69 patients at recruitment and at follow-up. In addition, a cardiovascular magnetic resonance substudy will be performed at follow-up in 35 patients. We will explore associations between cardiovascular magenetic resonance indexes of cardiac function, biomarkers of cardiac dysfunction and inflammation, and patient-reported outcome measures.

**Interpretation:**

CONDUCT-ICU will provide data regarding the cause and prevalence of cardiac dysfunction in survivors of sepsis and will explore associations with functional impairment. It will provide feasibility data and operational learning for larger studies investigating mechanisms of functional impairment after ICU admission and the association between sepsis and adverse cardiovascular events.

**Trial Registry:**

ClinicalTrials.gov; No.: NCT05633290; URL: www.clinicaltrials.gov

**Take-home Points**

**Study Question:** What is the prevalence of myocardial dysfunction after an ICU admission with sepsis and to what extent might it be associated with physical impairments in post-intensive care syndrome ?

**Methods:** The Characterisation of Cardiovascular Function in ICU Survivors of Sepsis (CONDUCT-ICU) study is a prospective, multicenter, multimodal cohort study combining cardiovascular magnetic resonance imaging, cardiac and inflammatory biomarkers, and patient-reported outcome measures in survivors of sepsis treated in the ICU.

**Interpretation:** CONDUCT-ICU will provide mechanistic data regarding the cause and prevalence of cardiac dysfunction after sepsis. In addition, we will gain key operational and logistical learning for future studies examining adverse cardiovascular outcomes and functional impairments after critical illness.

Sepsis is one of the most common reasons for ICU admission and mortality worldwide.^1^^,^^2^ However, for survivors the impact persists long after admission to hospital. Survivors of critical illness such as sepsis often experience significant physical, cognitive, or psychological impairments, often termed *post-intensive care syndrome* (PICS).^3^^,^^4^ However, the mechanisms by which these impairments occur after sepsis are less clear.^5^ Understanding mechanisms by which chronic impairments occur after initial recovery from acute illness is key to understanding how we can best improve care for survivors.

The adverse cardiovascular risk associated with sepsis has gained attention in recent years.^6^ A recent meta-analysis demonstrated an association with sepsis and adverse cardiovascular outcomes such as heart failure and myocardial infarction in a magnitude comparable with that of other major cardiovascular risk factors such as hypertension or dyslipidaemia.^7^ The postulated hypotheses that an acute inflammatory response to infection resulting in low-grade chronic inflammation and metabolic changes that predispose patients to atherosclerosis, hypertension, and ultimately chronic cardiovascular disease is compelling.^6^

The implications for survivorship are significant. Many survivors of critical illness such as sepsis experience symptoms such as breathlessness and fatigue.^8^^,^^9^ However, the extent to which cardiac dysfunction plays a role in these symptoms or the other functional impairments is unknown. Given the association of adverse cardiovascular events such as myocardial infarction or heart failure, it is plausible that survivors of sepsis may be at accelerated risk of chronic cardiovascular disease, and several mechanisms are at play that may be associated with increased risk of cardiovascular disease and functional impairment after critical illness (Fig 1).^10^

**Figure 1:**
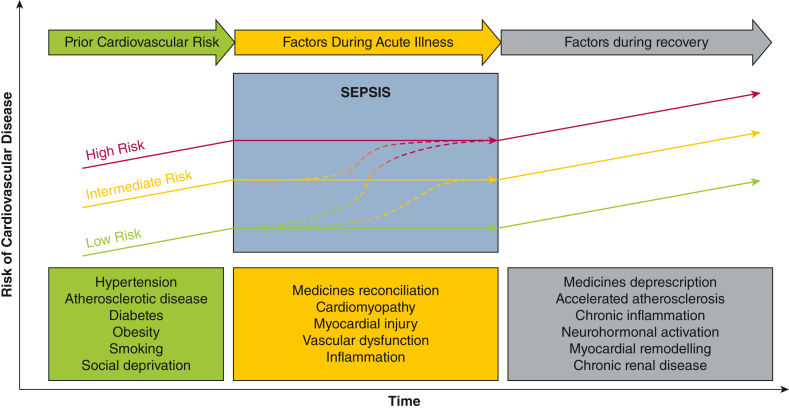
A model of cardiovascular risk after ICU admission with sepsis. Several potential factors related to premorbid risk, acute illness, and the recovery period may contribute toward or accelerate the risk of chronic cardiovascular disease following admission. We aim to explore these associations using a multimodal approach to help characterize cardiovascular dysfunction after admission with sepsis. (Adapted from Garrity et al.^10^)

To this end, Characterising Cardiac Function in ICU Survivors of Sepsis (CONDUCT-ICU) is a multicenter observational cohort study aimed at characterizing cardiac function in ICU survivors of sepsis and exploring the mechanisms by which it may contribute to functional impairments after admission. We address the question: What is the prevalence of myocardial dysfunction after ICU admission with sepsis and to what extent might it be associated with physical impairments in PICS?

## Study Design and Methods

### Objectives

This exploratory study will combine cardiovascular magnetic resonance (CMR) imaging, biomarkers of cardiac dysfunction and systemic inflammation, and patient-reported outcome measures. The study has six interrelated aims:

• To understand the feasibility of CMR in this complex, often frail patient cohort.
• To describe prevalence of left ventricular (LV) and right ventricular (RV) systolic dysfunction and to explore causative mechanisms (inflammation, scar, thrombus).
• To investigate biochemical changes associated with cardiac dysfunction and cardiac injury and how they compare with those in the general population.
• To describe the inflammatory profile of survivors of critical illness resulting from sepsis 6 to 10 weeks after hospital discharge.
• To explore the relationship between inflammation and chronic pain in survivors of critical illness resulting from sepsis.
• To explore the relationship among functional impairment, detailed imaging, and cardiac biomarkers.

### Study Design

CONDUCT-ICU is a prospective, multicenter, observational cardiovascular imaging and biomarker cohort study in survivors of sepsis treated in the ICU. CONDUCT-ICU has been approved by a National Health Service research and ethics committee (South-West Central Bristol; Identifier: 22/SW/0082). The study is sponsored by NHS Greater Glasgow and Clyde (Identifier: GN22CA029).

#### Population

Patients admitted to the ICU for sepsis, defined by internationally recognized Third International Consensus Definitions for Sepsis and Septic Shock definitions, will be considered for inclusion. Our study protocol is designed with a focus on patients receiving critical care during the convalescent phase of illness, and as such our findings will be most relevant to the population of survivors of intensive care.

**Figure 2:**
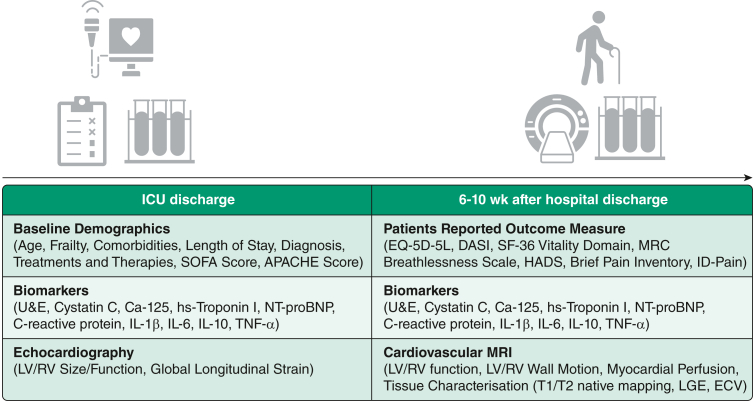
Diagram showing observations in the Characterisation of Cardiovascular Function in ICU Survivors of Sepsis study. APACHE = Acute Physiology and Chronic Health Evaluation; DASI = Dukes Activity Status Index; ECV = extracellular volume; HADS = Hospital Anxiety and Depression Index; LGE = late gadolinium enhancement; LV = left ventricle; MRC = Medical Research Council; NT-proBNP = N-terminal pro B-type natriuretic peptide; RV = right ventricle; SF-36 = 36-item Short-Form Health Survey; SOFA = Sequential Organ Failure Assessment; TNF-alpha = tumor necrosis factor alpha; U&E = urea and electrolytes.

After providing informed consent, patients will be recruited around the time of ICU discharge during which cardiac and inflammatory biomarkers and echocardiography were completed. Participants will be followed up 6 to 10 weeks after hospital discharge, when patient-reported functional outcome measures (PROMs) will be obtained, along with repeat cardiac and inflammatory biomarker analysis. A subgroup of patients will undergo echocardiography at baseline and CMR after discharge from hospital (Fig 2).

#### Setting

The participating study sites are two adult ICUs in the west of Scotland managing both medical and surgical patients. Imaging will be performed in a regional research center of excellence.

#### Eligibility Criteria

Eligibility criteria include provision of informed consent, age of 18 years or older, ICU admission with sepsis (according to the Third International Consensus Definitions for Sepsis and Septic Shock),^1^ and ability to comply with study procedures.

#### Exclusion Criteria

Exclusion criteria include inability to give informed consent, ongoing participation in any investigational research that may undermine the scientific basis of the study, receipt of immune modulating drugs or biologic therapy either long-term or during acute admission, patients considered by the clinical team to be very unlikely to survive to hospital discharge, hospital admission because of COVID-19, patients undergoing treatment for malignancy with systemic anticancer therapies, pregnancy, and patients who are prisoners at time they otherwise would be eligible.

Participants will be excluded from the CMR cohort if any of the following are present: any contraindication to MRI, known coronary artery disease before ICU admission, previous myocardial infarction before ICU admission, and known chronic heart failure before ICU admission.

#### Screening

We aim to screen most patients admitted to the ICU with sepsis over the course of a 15-month period. The reasons for ineligibility will be recorded prospectively to characterize any selection bias.

#### Recruitment

Patients who are identified as being ready for discharge from intensive care by their local clinical care team will be identified if eligible and approached by recruiters. Sixty-nine patients will be recruited for the study in total. Within this study cohort, a subgroup of 35 patients will undergo echocardiography at baseline and CMR at 6 to 10 weeks. All patients eligible for recruitment to CMR investigation as outlined by the exclusion criteria will be recruited to this arm to minimize selection bias until the scanning phase of the study is complete. We will collect cardiac and inflammatory biomarker levels in all patients and will explore relationships to impairments such as reduced functional capacity or chronic pain. Consent will include longer-term follow-up and storage of biological samples for future analysis, pending further ethical approval.

#### Diagnosis of Sepsis

The diagnosis of sepsis will be in accordance with Third International Consensus Definitions for Sepsis and Septic Shock definitions.^1^ These are internationally recognized and validated definitions with widespread use in critical care research and clinical practice.^1^^,^^11^ Organ dysfunction will be defined as a change in Sequential Organ Failure Assessment score of ≥ 2.^1^

### Outcomes

#### Primary Outcome and Justification

The primary outcome for the imaging component of the study is prevalence of reduced left ventricular ejection fraction (LVEF) in the sample population at 6 to 10 weeks after hospital discharge. Reduced LVEF will be considered < 51% for females and < 47% for males, aligned with local control population and within parameters advocated by the European Association for Cardiovascular Imaging.^12^^,^^13^

LVEF is fundamental to the assessment of cardiac dysfunction and has been used widely in patients with heart failure.^14^ Along with its inclusion in international guidelines, a well-established evidence base exists for treatment of heart failure with reduced ejection fraction.^14^ Therefore, it represents a clinically important outcome for this population that may have an impact on mortality, quality of life, or both.

Using gold standard imaging techniques such as CMR in combination with biomarker analysis is novel in this population and will provide objective measures of both inflammation and cardiac function that can be compared with those in other populations such as healthy control participants or other vulnerable patient groups. CMR and biomarker analysis has been used in our institution in a variety of settings, including pulmonary hypertension, COVID-19, after lung resection surgery, and after myocardial infarction.^15^, ^16^, ^17^ Given that sepsis is associated with an increased incidence of heart failure in survivors, CMR represents an important technique for advancing our understanding and providing clinically important data that can be referenced and compared with other populations.^7^^,^^18^

#### Secondary Outcomes

In addition to examining the prevalence of reduced LVEF, we will evaluate a series of other relevant exploratory outcomes. CMR offers the ability to explore native T1 and T2 mapping, markers of subtle fibrosis and edema for which abnormal values have been identified in other populations.^19^^,^^20^

We will explore other measures of cardiac injury or dysfunction during the index illness in patients who undergo CMR. In addition to collection of cardiac biomarkers of injury or dysfunction on ICU discharge (troponin and N-terminal pro B-type natriuretic peptide), we will evaluate for evidence of myocardial injury during ICU admission. We will record evidence of any significant new arrhythmia or myocardial injury documented on 12-lead ECG during admission or significant arrhythmia on three-lead monitoring and documented by the clinical care team. We will record any echocardiography findings during index ICU admission. Many patients with sepsis and septic shock will undergo ECG in the ICU for assessment and refinement of diagnoses or to tailor supportive therapies.^21^ ECG involves use of noninvasive ultrasound imaging to acquire measurements of heart structure and function. It is undertaken commonly in a focused manner by clinicians in the ICU, and these images can be analyzed retrospectively and can be correlated with other clinically meaningful end points.^21^^,^^22^ Ensuring that we can correlate CMR findings with ECG during acute illness will allow us to triangulate multiple data points and methods of cardiovascular assessment over the course of participant follow-up.

#### Patient-Reported Outcome Measures

To examine functional impairment, PROMs and their association with findings on cardiac imaging and biomarkers will be explored. These will include established PROMs used in the assessment of functional capacity and breathlessness: the Duke Activity Status Index, the Medical Research Council dyspnea scale, and the EQ-5D-5L assessment.^23^, ^24^, ^25^, ^26^, ^27^ To assess outcomes pertaining to fatigue, the vitality domain of the 36-item Short-Form Health Survey questionnaire will be collected.^28^^,^^29^ The Brief Pain Inventory (BPI), ID Pain, and the Hospital Anxiety and Depression Score will allow the relationship among pain, mental health outcomes, and biomarkers of inflammation to be assessed.^30^, ^31^, ^32^

#### Biomarkers

In addition to biomarkers of myocardial injury or dysfunction (high-sensitivity troponin and N-terminal pro B-type natriuretic peptide), we will collect biomarkers of inflammation on ICU discharge and at 6 to 10 weeks after hospital discharge (C-reactive protein, IL-1β, IL-6, tumor necrosis factor alpha, and IL-10). These biomarkers have been associated with adverse cardiovascular events, allowing us to explore the mechanistic underpinnings of observed cardiovascular outcome measures.^33^^,^^34^ Furthermore, embedded within our study, we plan to explore other mechanisms of adverse outcomes such as chronic pain. Chronic pain can have a profound impact on quality of life, and our biomarker analysis offers an opportunity to study pathologic mechanisms that may be implicated in development of chronic pain after discharge.^35^ Biomarkers will be batch analyzed at University of Glasgow laboratories.

### Cardiovascular MRI

CMR is a gold standard method for assessing indices of cardiac function and a reference diagnostic method for assessing myocardial injury, including myocarditis and cardiomyopathy,^18^^,^^36^^,^^37^ often with superior accuracy and precision when compared with ECG.^36^^,^^37^ Thus, it can be used as a reference method to assess myocardial function, tissue characterization, and presence of scar.

CMR will be conducted using a 3.0-Tesla (Siemens PRISMA) scanner 6 to 10 weeks after discharge by a Health and Care Professions Council-accredited radiographer. Postprocessing will be protocolized and dual-reported. The 6- to 10-week time point coincides with the point at which participating sites routinely follow-up survivors of critical illness and as such, is the point at which we normally would evaluate and address ongoing comorbidity after critical care admission.^38^^,^^39^ Although it is possible to detect myocardial inflammation earlier than this in other patient cohorts, we wish to minimize the burden on patients in terms of follow-up at multiple appointments and to ensure that we identify findings at a time point when they are likely to have meaningful implications for patient recovery.

Sepsis-induced cardiomyopathy may occur in 10% to 70% of patients admitted with sepsis. Traditionally, it has been thought to be reversible, with a recovery of LV function; however, we now know that sepsis is associated with an adverse risk of heart failure or myocardial infarction.^40^ In other settings such as LV assist device insertion, persistent molecular abnormalities persist even after cardiac function has improved.^41^ CMR will allow us to determine if new or persisting myocardial dysfunction, myocarditis, or new incidental pathologic features (eg, mural thrombus) are present. Furthermore, we will identify any incidental or unexpected pathologic features (eg, pulmonary arterial thrombus or incidental tumor).

### Feasibility

The feasibility of CMR in this population is of great significance to further mechanistic studies in the population of ICU survivors. To our knowledge, imaging of this type has never been undertaken prospectively in this population in combination with PROMS and biomarker analysis. Demonstrating feasibility of cardiovascular MRI in survivors of an ICU stay will pave the way for larger studies examining mechanisms of functional impairment in the population receiving critical care.

### Statistical Considerations

#### Sample Size Calculation for Cardiac Imaging

Given the exploratory nature of this study and the paucity of data available for CMR outcomes in a population after critical illness, it is difficult to undertake an accurate power calculation for prevalence of LV dysfunction. Nonetheless, we will explore the usefulness of CMR for assessing cardiac dysfunction in this patient cohort. We will use CMR data to explore the impact of sepsis on LV function, cardiac inflammation, and other parameters such as strain.^19^

Patients with sepsis in the ICU show a wide-ranging prevalence of LV dysfunction (10%-63%).^42^, ^43^, ^44^ If a conservative estimate of 12% prevalence of LV dysfunction after ICU admission is taken, and ensuring 80% power with a significance of 0.05, then recruitment of 35 patients for imaging, biomarkers, and PROMS would be required to determine a significant difference when compared with population control participants in whom prevalence is 2.2%.^45^

#### Sample Size for Biomarkers

Biomarkers of cardiac function will be assessed in all participants in this study in combination with markers of inflammation. We will conduct exploratory analysis to explore the association between markers of inflammation (eg, IL-6) and other physical impairments experienced after ICU admission, such as pain. Pain scores will be assessed using the severity of pain question set of the BPI (where any response of > 0 indicates that at least mild pain in present).

**Figure 3:**
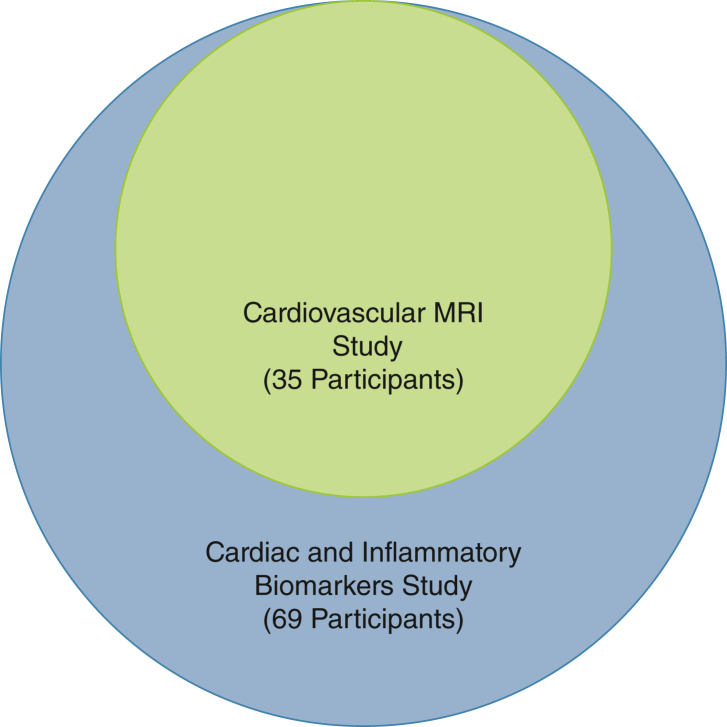
Venn diagram showing structure of recruitment for the Characterisation of Cardiovascular Function in ICU Survivors of Sepsis study. Participants in the green area will undergo CMR, biomarker analysis, and patient-reported outcome measure analysis. Participants in the blue area will undergo patient-reported outcome measure and biomarker analysis only.

Using data collected from a previous local study investigating the incidence of chronic pain in survivors of critical illness, the mean BPI score was 6.5 (SD, 2.63) at 3 months.^46^ No studies are available in the critical care field that have investigated inflammation and pain longitudinally across the recovery trajectory. As such, we used data describing the association between inflammatory markers and chronic back pain, which found that a two-point reduction in 10-point pain severity scale was associated with lower IL-6 levels.^47^ Given the heterogeneity of the critical care population, we opted for a conservative estimate of a one-point change in BPI score with an α of .05 and power of 80%.^48^ As such, a sample size of 69 patients (Fig 3) will be recruited for biomarker analysis, allowing for a 20% attrition rate (resulting from loss to follow-up and mortality). This sample size also will allow for detection differences in cardiac biomarkers such as N-terminal pro B-type natriuretic peptide seen in other studies with at least 80% power and a significance level of 0.05.^20^

#### Trial Management and Timelines

This study will be conducted in line with the current Guidelines for Good Clinical Practice in Clinical Trials and Strengthening the Reporting of Observational Studies in Epidemiology statement guidelines.^49^ The Study Management Group includes individuals responsible for the day-to-day management of the study, including the chief investigator (T. Q.), representatives from the sponsor, and the principal investigators at the designated study sites (K. G., C. D.). This group will ensure adherence to the protocol and will take appropriate action to safeguard participants and the quality of the study itself. Decisions about continuation or termination of the study will be the responsibility of the sponsor.

## Discussion

CONDUCT-ICU is a prospective, observational, multisite imaging and biomarker cohort study of survivors of sepsis treated in the ICU aimed at characterization of cardiovascular function and functional impairments after admission. To our knowledge, the approach is novel in this population, allowing us to explore potential mechanisms that mediate adverse cardiovascular events associated with sepsis admission. CONDUCT-ICU will provide objective measurements of cardiac function, myocardial inflammation, edema, and scar, and these findings will be enhanced by the ability to explore associations with cardiac and inflammatory biomarkers in addition to PROMs.

Critical illnesses such as sepsis are associated with chronic immune activation and immunometabolic changes similar to those seen in chronic cardiovascular disease.^6^ These inflammatory and metabolic changes can result in atherosclerosis or cardiac remodelling that ultimately may predispose patients to hypertension, ischemic heart disease, or heart failure. Survivors of ICU admission represent a large population of patients at potential risk of chronic cardiovascular disease. CONDUCT-ICU will allow us to collate evidence prospectively to help support or refute proposed clinical mechanisms and to identify a group of patients who may be responsive to established therapeutics. Furthermore, we also will be able to explore other impairments such as chronic pain through the larger cohort of patients undergoing biomarker analysis.

We will recruit a deliberately heterogenous cohort of patients in terms of comorbidity, admission diagnosis, and hospital trajectory. In doing so, we will minimize selection bias and ensure that the findings will be generalizable. ICU admission has a great impact on patient quality of life, with nearly one-third of patients readmitted to the hospital within 90 days and almost one-half struggling to return to work within 1 year.^50^ As such, it is possible that we will lose a significant proportion of patients to follow-up. CONDUCT-ICU will inform attrition rates, sample sizes, and feasibility of outpatient investigations in this population. Incorporating the imaging component of CONDUCT-ICU into a wider cohort of patients undergoing biomarker analysis will allow us to minimize attrition from this arm of the study.

We recognize that this study has limitations. Although we are making efforts to exclude patients with established coronary artery disease or heart failure, we recognize that some patients may have unrecognized cardiovascular disease at the point of recruitment to the study, potentially confounding the results. Our methodology represents a pragmatic approach to recruiting in this patient cohort, balancing exclusion of confounders and avoidance of exposure to the burden of additional imaging or invasive angiography during what often is a prolonged and complicated stay. Exclusion of patients unable to give consent may result in selection bias, for example, through excluding patients because of delirium that has not yet resolved. Inclusion of these patients via other means such as proxy consent would present logistical and ethical challenges on follow-up, and as such we have excluded these patients from this initial feasibility study.

CONDUCT-ICU is a novel multimodal imaging and biomarker study that will give fundamental mechanistic insights into adverse cardiovascular outcomes after ICU admission with sepsis. It will provide key understanding into the role cardiovascular dysfunction may play in functional impairment after ICU care. Furthermore, it will provide key operational and pilot data for larger studies investigating mechanisms of functional impairments occurring after ICU care in sepsis and other critical illness.

## Funding/Support

CONDUCT-ICU is funded in part via a Wellcome Trust Translational Partnership Award [Grant 219390/Z/19/Z]. Biomarker analysis is supported by the Fiona Elizabeth Agnew Trust. K. G. and C. D. are funded through clinical research fellow posts at the University of Glasgow.

## Financial/Nonfinancial Disclosures

None declared.
